# Impact of chemical reaction on the Cattaneo–Christov heat flux model for viscoelastic flow over an exponentially stretching sheet

**DOI:** 10.1038/s41598-024-65642-9

**Published:** 2024-07-11

**Authors:** Abdelmgid O. M. Sidahmed, Faisal Salah, K. K. Viswanathan

**Affiliations:** 1 Department of Mathematics, College of Science & Arts, King Abdulaziz University, 21911 Rabigh, Saudi Arabia; 2 Department of Mathematical Modeling, Faculty of Mathematics, Samarkand State University, 15, University Blvd., 140104 Samarkand, Uzbekistan

**Keywords:** Chemical reaction, Cattaneo–Christov heat flux, Upper-convected maxwell fluid, Successive linearization method, Exponentially stretching sheet, Engineering, Mathematics and computing

## Abstract

In this article, the numerical solutions for the heat transfer flow of an upper-convected Maxwell fluid across an exponentially stretched sheet with a chemical reaction on the Cattaneo–Christov heat flux model have been investigated. Using similarity transformation, the controlling system of nonlinear partial differential equations was transformed into a system of ordinary differential equations. The resulting converted equations were solved numerically by a successive linearization method with the help of MATLAB software. A graphic representation was created to analyze the physical insights of the relevant flow characteristics. The findings were presented in the form of velocity, temperature, and concentration profiles. As the relaxation time parameter varied, the local Nusselt number increased. The thermal relaxation time was shown to have an inverse relationship with fluid temperature. Furthermore, the concentration boundary layer becomes thinner as the levels of the reaction rate parameter increase. The results of this model can be applicable in biological fluids and industrial situations. Excellent agreement exists between the analysis's findings and those of the previous studies.

## Introduction

The laminar flow and heat transfer past a stretching sheet have many industrial uses and impact technological processes. In previous studies, it was assumed that the velocity of a stretched surface is linearly proportional to its distance from a fixed origin. Fourier’s^1^ proposed law on heat conduction has been used as a basis for predicting heat transfer behavior in a variety of real-world contexts. Cattaneo^2^ modified Fourier’s law to include the relaxation time for heat flux, which is the amount of time required to achieve constant heat conduction after the imposition of a temperature gradient. Straughan^3^ examined the thermal convection in an incompressible flow using the Cattaneo–Christov model. Ciarletta and Straughan^4^ showed that the Cattaneo–Christov equations are stable and unique. To account for the Cattaneo–Christov heat flux, Mustafa^5^ constructed analytical and numerical solutions for rotating Maxwell fluid flow. They found that the Prandtl number and relaxation period for the heat flux are inversely correlated with the fluid temperature. Numerous industrial applications of non-Newtonian fluids with convective heat and mass transfer include the flow of biological fluids, coatings for paper and liquid metals, plastic extrusion, material processing, and crystal growth. Using the Cattaneo–Christov heat flux model, Khan et al.^6^ investigated the boundary layer flow of an upper-convected Maxwell fluid (UCM) caused by an exponentially extending sheet. They discovered that the fluid temperature and thermal relaxation time are inversely related. The fluid velocity further decreased as the fluid relaxation time increased. By applying the Cattaneo–Christov model, Sohail and Naz^7^ investigated the Sutterby nanofluid MHD flow for heat and mass diffusion. Williamson studied MHD nanofluid flow via a stretchable plate using fractional Cattaneo–Christov heat theory by Khan and Alzahrani^8^. Dadheech et al.^9^, investigated numerical study of entropy generation on Williamson fluid across a permeable vertical plate along with non-liner chemical reaction as well as slip condition. They found that entropy generation rate enhances for higher values of Brinkman number. Recent research that considers the Cattaneo–Christov theory can be found in^10–15^.

Chemical reactions are interactions that occur between substances to create new substances of various chemical compositions. A chemical reaction is a process by which reactants are converted into products^16,17^. Seini and Makinde^18^ investigated how the MHD boundary layer moved across an exponentially stretched sheet in the presence of chemical reactions and radiation. By employing the Bvp4c method, Paul and Kanti^19^ studied a two-dimensional stability issue that integrates the magnetohydrodynamic effect with three separate flows of fluid from the boundary layer across an exponentially stretched sheet under the impact of thermal radiation and chemical reactions. The shooting method^20,21, 22^, Keller box method^23^, finite element method^24,25^, homotopy perturbation method^26^ and bvp4c^27,28–30^ are numerical methods used to handle some of these issues. The successive linearization method (SLM) has recently been employed by many researchers. The controlling nonlinear equations were transformed using this method into a set of linear differential equations. We applied the Chebyshev pseudo-spectral method to resolve the higher-order deformation in the linear differential equations. According to the Chebyshev spectral collocation differentiation matrix presented in Makukula et al.^31^, an auxiliary linear operator is defined. Ahmed et al.^32^ applied the successive linearization method to study the effects of radiation and viscous dissipation on MHD boundary layer convective heat transfer with low pressure gradient in porous media. Khidir^33^ applied the successive linearization method on the nonlinear boundary value problem of MHD boundary layer analysis for heat and mass transfer. In comparison to other current semi-analytical approaches, such as the Adomian decomposition method, they demonstrated that the SLM swiftly converges to numerical values and is flexible, efficient, and accurate. The SLM approach can also be used to handle boundary value problems involving highly nonlinear systems, instead of more traditional numerical approaches (see references^34,35^).

The studies mentioned above revealed that no research on the effect of chemical reaction on the Cattaneo–Christov heat flux model for viscoelastic flow over an exponentially stretching sheet has been done. The prime motivation of our analysis is to expand on the findings of Khan et al.^6^, such as how the Cattaneo–Christov convection flow model affects the viscoelastic flow caused by a chemical reaction-filled slab that grows at an exponential rate. However, because it has numerous applications in chemical and manufacturing processes, such as polymer extrusion, continuous metal casting, copper wire extrusion, die forging, paper production, and many more, the study of viscous flow and heat transfer above stretching surfaces has received a lot of attention. Tables and graphs are used in this study to show the effects of various parameters found in the governing equations. We applied the SLM approach to numerically handle this problem, using a more efficient calculation. Quantitative investigation and plotting of pertinent results.

The rest of the paper is organized as follows. The governing system of nonlinear partial differential equations has been converted into a system of ordinary differential equations in Section "Problem formulation". Section "Numerical methods" deals with the application of SLM to solve our problem. Finally, some numerical results along with a discussion on them are given in Section "Results and discussion".

## Problem formulation

**Figure 1:**
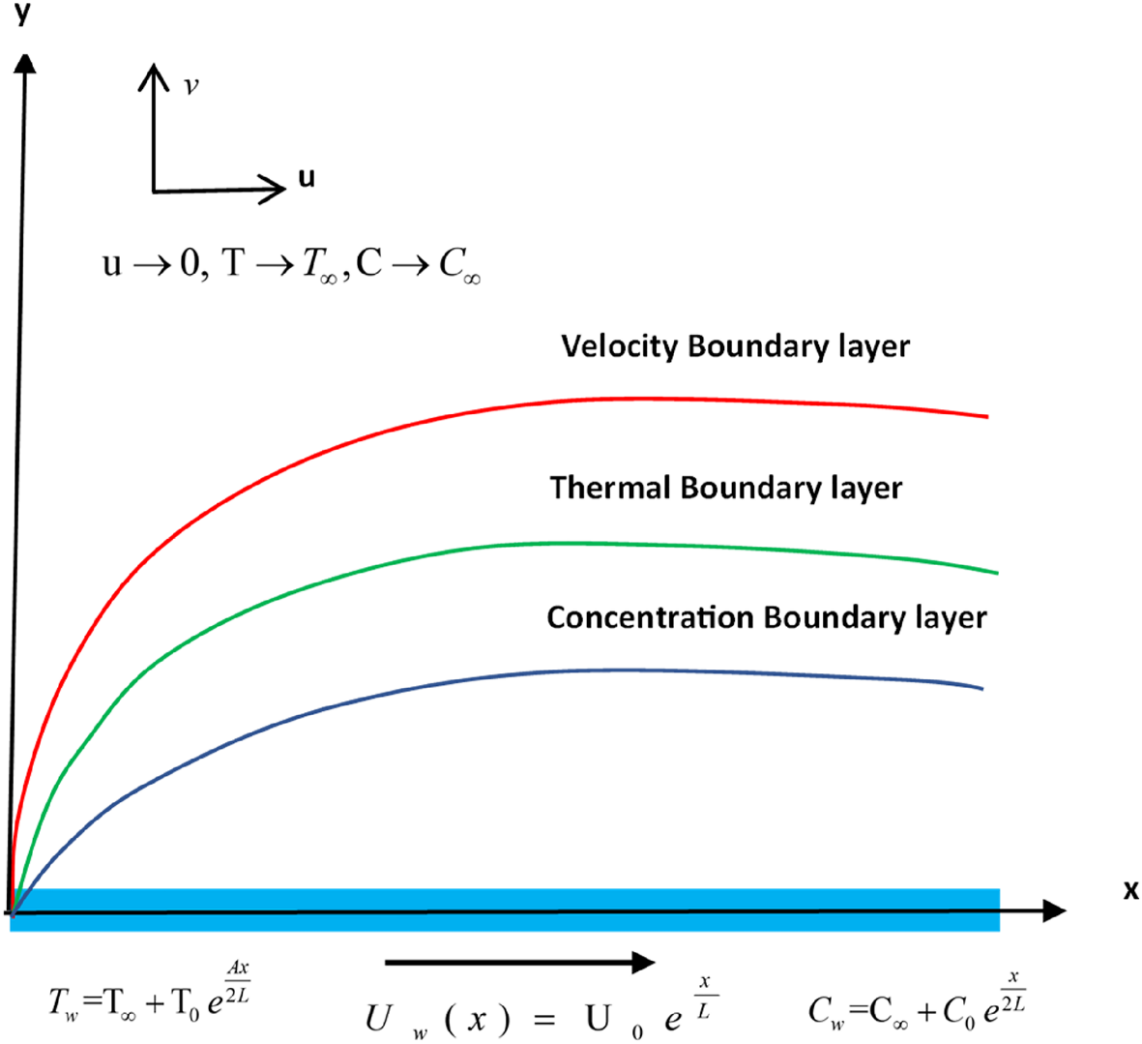
Physical model and coordinate system.

Consider an upper-convected Maxwell fluid (UCM) that flows incompressible in two dimensions across an elastic sheet during $y = 0$ (see Fig. 1). Applying equal and opposite forces along the $x -$ axis and considering that the flow is bound to the region where $y > 0$ occurs, the flow is generated as a result of the stretching surface. At time $t = 0$, unsteady fluid and mass flows begin. The sheet emerges from the origin through a slit and flows at the velocity of the $U_{w} (x) = {\text{U}}_{0} \,e^{\frac{x}{L}}$. The heating/cooling reference temperature ${\text{T}}_{0}$ is denoted by the variable surface temperature distribution $T_{w} {\text{ = T}}_{\infty } \, + {\text{T}}_{0} \,e^{{\frac{Ax}{{2L}}}}$(Magyari and Keller^36^), and mass concentration $C_{w} {\text{ = C}}_{\infty } \, + C_{0} \,e^{\frac{x}{2L}}$( Reddy et al.^37^), which is considered.

The formulation of the present problem is modelled with respect to following presumptions:

1. Upper-convected Maxwell fluid (UCM) flow
2. Micropolar liquid model
3. Cattaneo–Christov Heat Flux Model
4. Thermophoresis and chemical reaction effects are considered

Under the above assumptions, the governing equations so obtained are given by Khan et al.^6^

1 $$\nabla \cdot {\varvec{V}} = 0,$$

2 $$\rho {\varvec{a}} = \nabla \cdot {\varvec{T}},$$

where ${\varvec{V}}$ is the velocity vector, ${\varvec{T}}$ is the Cauchy stress tensor and ${\varvec{a}}$ is acceleration vector given by:

3 $$\user2{a = }\frac{{d{\varvec{V}}}}{dt} = \frac{{\partial {\varvec{V}}}}{\partial t} + \left( {{\varvec{V}} \cdot \nabla } \right){\varvec{V}}.$$

The Cauchy stress tensor for a Maxwell fluid is:

4 $${\varvec{T}} = - \rho \user2{I + S},$$

where the extra stress tensor ${\varvec{S}}$ satisfies

5 $${\varvec{S}}\, + \lambda_{1} \left( {\frac{{d{\varvec{S}}}}{dt} - {\varvec{LS}}\, - {\varvec{SL}}^{T} } \right) = \mu {\varvec{a}}_{1} ,$$

in which $\mu$ is the viscosity, $\lambda_{1}$ is the relaxation time, ${\varvec{L}}$ is the velocity gradient, and the Rivlin–Ericksen tensor ${\varvec{a}}_{1}$ is defined through

6 $${\varvec{a}}_{1} = {\varvec{L}} + {\varvec{L}}^{T} .$$

For a two-dimensional flow having velocity ${\varvec{V}}$ one gets in the absence of pressure gradient the following equations in component form

7 $$\rho \left[ {u\frac{\partial u}{{\partial x}} + v\frac{\partial u}{{\partial y}} + \lambda_{1} \left\{ {u^{2} \frac{{\partial^{2} u}}{{\partial x^{2} }} + v^{2} \frac{{\partial^{2} u}}{{\partial x^{2} }} + 2uv\frac{{\partial^{2} u}}{\partial x\partial y}} \right\}} \right] = \mu \left( {\frac{{\partial^{2} u}}{{\partial x^{2} }} + \frac{{\partial^{2} u}}{{\partial y^{2} }}} \right),$$

8 $$\rho \left[ {u\frac{\partial v}{{\partial x}} + v\frac{\partial v}{{\partial y}} + \lambda_{1} \left\{ {u^{2} \frac{{\partial^{2} v}}{{\partial x^{2} }} + v^{2} \frac{{\partial^{2} v}}{{\partial x^{2} }} + 2uv\frac{{\partial^{2} v}}{\partial x\partial y}} \right\}} \right] = \mu \left( {\frac{{\partial^{2} v}}{{\partial x^{2} }} + \frac{{\partial^{2} v}}{{\partial y^{2} }}} \right),$$

9 $$\frac{\partial u}{{\partial x}} + \frac{\partial v}{{\partial y}} = 0.$$

Using the boundary layer approximations^38^

10 $$u = o(1),\,\,v = o(\delta ),\,\,x = o(1),\,\,y = o(\delta ),$$

where $\delta$ being the boundary layer thickness, the flow is governed by Eq. (9) and

11 $$u\frac{\partial u}{{\partial x}} + v\frac{\partial u}{{\partial y}} + \lambda_{1} \left( {u^{2} \frac{{\partial^{2} u}}{{\partial x^{2} }} + v^{2} \frac{{\partial^{2} u}}{{\partial y^{2} }} + 2uv\frac{{\partial^{2} u}}{\partial x\partial y}} \right) = \upsilon \frac{{\partial^{2} u}}{{\partial y^{2} }},$$

12 $$(\rho c_{P} )\left( {u\frac{\partial T}{{\partial x}} + v\frac{\partial T}{{\partial y}}} \right) = - \nabla .\,\,q,$$

13 $$u\frac{\partial C}{{\partial x}} + v\frac{\partial C}{{\partial y}} = D\frac{{\partial^{2} C}}{{\partial y^{2} }} - k_{1} (x)\left( {C - C_{\infty } } \right).$$

where $u$ and $v$ represent the velocity's $x -$ and $y -$ directional components, respectively. The following relationship^39^ holds when $\upsilon$ is the kinematic viscosity,$\lambda_{1}$ is the fluid relaxation time, $T$ is the local fluid temperature, $k_{1} (x)$ is the chemical reaction rate, and $q$ is the heat flux which satisfies the following relationship

14 $$q + \lambda_{2} \left( {\frac{\partial q}{{\partial t}} + V.\nabla q - q.\nabla \,V + (\nabla .\,\,V)\,q} \right) = - k\nabla \,T,$$

where $V$ is the velocity vector, $k$ is the thermal conductivity, and $\lambda_{2}$ is the heat-flow relaxation time. We arrive at the following equations after eliminating $q$ from Eqs. (3) and (4) (see Han et al.^40^ and Christov^41^).

15 $$u\frac{\partial T}{{\partial x}} + v\frac{\partial T}{{\partial y}} + \lambda_{2} \left[ {\left( {u\frac{\partial u}{{\partial x}} + v\frac{\partial u}{{\partial y}}} \right)\frac{\partial T}{{\partial x}} + \left( {u\frac{\partial v}{{\partial x}} + v\frac{\partial v}{{\partial y}}} \right)\frac{\partial T}{{\partial y}}} \right.\left. { + u^{2} \frac{{\partial^{2} T}}{{\partial x^{2} }} + v^{2} \frac{{\partial^{2} T}}{{\partial y^{2} }} + 2uv\frac{{\partial^{2} T}}{\partial x\partial y}} \right] = \alpha \frac{{\partial^{2} T}}{{\partial y^{2} }},$$

where $\alpha ( = k/\rho c_{P} )$ is the thermal diffusivity.

### boundary conditions on velocity

The boundary sheet is assumed to be stretched with a large force in such a way that stretching velocity along the axial direction $x$ is of exponential order of the directional coordinate. Hence, we employ the following boundary conditions on velocity (see Khan et al.^6^).

16 $$\begin{gathered} u = U_{w} = {\text{U}}_{0} \,e^{\frac{x}{L}} ,\,\,v = 0, \hfill \\ T = T_{w} = T_{\infty } + T_{0} \,e^{{\frac{Ax}{{2L}}}} , \, \hfill \\ {\text{C = C}}_{w} {\text{ = C}}_{\infty } {\text{ + C}}_{0} \,e^{\frac{x}{2L}} \, at \, y = 0. \hfill \\ u \to 0,T \to T_{\infty } ,{\text{ C }} \to {\text{ C}}_{\infty } \, at \, y \to \infty . \hfill \\ \end{gathered}$$

Using the similarity transformations shown below^36^

17 $$\begin{gathered} \eta = y\sqrt {\frac{{U_{0} }}{2\upsilon L}} \,e^{{{x \mathord{\left/ {\vphantom {x {2L}}} \right. \kern-0pt} {2L}}}} , \hfill \\ u = U_{0} e^{{{x \mathord{\left/ {\vphantom {x L}} \right. \kern-0pt} L}}} f^{\prime}(\eta ), \hfill \\ v = - \sqrt {\frac{{\upsilon U_{0} }}{2L}} e^{{{x \mathord{\left/ {\vphantom {x {2L}}} \right. \kern-0pt} {2L}}}} \left[ {f(\eta ) + \eta f^{\prime}(\eta )} \right], \hfill \\ T = T_{\infty } + T_{0} \,e^{{{{Ax} \mathord{\left/ {\vphantom {{Ax} {2L}}} \right. \kern-0pt} {2L}}}} \theta (\eta ), \hfill \\ C = C_{\infty } + C_{0} \,e^{{{x \mathord{\left/ {\vphantom {x {2L}}} \right. \kern-0pt} {2L}}}} \phi (\left( \eta \right). \hfill \\ \end{gathered}$$

We see that similarity exists by substituting Eq. (17) into Eqs. (11) – (16), and we obtain the following:

18 $$f^{\prime\prime\prime} - 2f^{{\prime}{2}} + ff^{\prime\prime} + \Lambda_{1} \left( {3ff^{\prime}f^{\prime\prime} + \frac{\eta }{2}f^{{\prime}{2}} f^{\prime\prime} - \frac{1}{2}f^{2} f^{\prime\prime\prime} - 2f^{{\prime}{3}} } \right) = 0,$$

19 $$\frac{1}{\Pr }\theta^{\prime\prime} + f\theta^{\prime} - Af^{\prime}\theta + \frac{{\Lambda_{2} }}{2}\left[ {Aff^{\prime\prime}\theta - A(2 + A)f^{{\prime}{2}} \theta } \right.\left. { + (1 + 2A)ff^{\prime}\theta^{\prime} - f^{2} \theta^{\prime\prime}} \right] = 0,$$

20 $$\phi^{\prime\prime} + S_{c} \left( {f\phi^{\prime} - f^{\prime}\phi - \gamma \phi } \right) = 0,$$

21 $$\begin{gathered} f(0) = 0,\,\,f^{\prime}(0) = 1,\,\,\theta (0) = 1,\,\,\phi (0) = 1, \hfill \\ f^{\prime}(\infty ) \to 0,\,\,\theta (\infty ) \to 0,\,\,\phi (\infty ) \to 0\,\,, \hfill \\ \end{gathered}$$

where $$\Pr = \frac{\upsilon }{\alpha }$$ is the Prandtl number, $$S_{c} = \frac{\upsilon }{D}$$ is the Schmidt number, $$\gamma$$ is the reaction rate parameter, and $$\Lambda_{1} = \frac{{\lambda_{1} U_{0} e^{{{\raise0.7ex\hbox{$x$} \!\mathord{\left/ {\vphantom {x L}}\right.\kern-0pt} \!\lower0.7ex\hbox{$L$}}}} }}{L}$$,$$\Lambda_{2} = \frac{{\lambda_{2} U_{0} e^{{{\raise0.7ex\hbox{$x$} \!\mathord{\left/ {\vphantom {x L}}\right.\kern-0pt} \!\lower0.7ex\hbox{$L$}}}} }}{L}$$ is the non-dimensional fluid relaxation time and thermal relaxation time. The case of a Newtonian fluid is achieved when $$\Lambda_{1} = 0$$ in Eqs. (18)–(21). In addition, $$\Lambda_{2} = 0$$ fits the original Fourier law of heat conduction.

The skin friction coefficient $C_{f}$ defined as:

$$\frac{1}{\sqrt 2 }C_{f} \sqrt {\text{Re}} = f^{\prime\prime}(0).$$

The heat and mass transfers from the plate, respectively, are given by

$$q_{w} = - k\left( {\frac{\partial T}{{\partial y}}} \right)_{y = 0} ,\;q_{m} = - D\left( {\frac{\partial C}{{\partial y}}} \right)_{y = 0} ,\;\frac{{N_{u} }}{{\sqrt {{\text{R}}_{e} } }} = - \theta^{\prime}(0),\;\frac{{S_{h} }}{{\sqrt {{\text{R}}_{e} } }} = - \phi^{\prime}(0),{\text{where}}\,\,\,R_{e} = \frac{{U_{w} }}{\upsilon }$$

## Numerical methods

We employed SLM to solve the current problem numerically using MATLAB script file code. The SLM works by iteratively converting the controlling nonlinear Eqs. (18) - (20) into a set of linear differential equations, which are then solved either analytically or numerically.

The SLM technique presupposes that the solutions of systems (18)–(20) can be represented as ^31,42^

22 $$f(\eta ) = f_{i} (\eta ) + \sum\limits_{n = 0}^{i - 1} {f_{n} (\eta )} ,\,\,\,\,\theta (\eta ) = \theta_{i} (\eta ) + \sum\limits_{n = 0}^{i - 1} {\theta_{n} (\eta )} ,\,\,\,\phi (\eta ) = \phi_{i} (\eta ) + \sum\limits_{n = 0}^{i - 1} {\phi_{n} (\eta )} .\,\,$$

Starting from an initial guess that is appropriate for $f_{0} (\eta ),\,\,\theta_{0} (\eta )$ and $\phi_{0} (\eta )$ and satisfies boundary conditions (21), suitable functions are as follows:

23 $$f_{0} (\eta ) = 1 - e^{ - \eta } ,\,\,\theta_{0} (\eta ) = e^{ - \eta } ,\,\,\phi_{0} (\eta ) = e^{ - \eta } .$$

Substituting Eq. (22) into controlling Eqs. (18) – (20) while neglecting the nonlinear factors in $f_{i} (\eta ),\,\,\theta_{i} (\eta )$ and $\phi_{i} (\eta )$ and their derivatives yields

24 $$\begin{gathered} \left( {1 - \frac{{\Lambda_{1} }}{2}\left( {\sum\limits_{j = 0}^{i - 1} {f_{j} } } \right)^{2} } \right)f^{\prime\prime\prime}_{i} + \left( {\sum\limits_{j = 0}^{i - 1} {f_{j} } + \frac{\eta }{2}\Lambda_{1} \left( {\sum\limits_{j = 0}^{i - 1} {f^{\prime}_{j} } } \right)^{2} + 3\Lambda_{1} \sum\limits_{j = 0}^{i - 1} {f_{j} } \sum\limits_{j = 0}^{i - 1} {f^{\prime}_{j} } } \right)f^{\prime\prime}_{i} \hfill \\ + \left( {3\Lambda_{1} \sum\limits_{j = 0}^{i - 1} {f_{j} } \sum\limits_{j = 0}^{i - 1} {f^{\prime\prime}_{j} + \eta \Lambda_{1} \sum\limits_{j = 0}^{i - 1} {f^{\prime}_{j} } \sum\limits_{j = 0}^{i - 1} {f^{\prime\prime}_{j} - 6} } \Lambda_{1} \left( {\sum\limits_{j = 0}^{i - 1} {f^{\prime}_{j} } } \right)^{2} - 4\sum\limits_{j = 0}^{i - 1} {f^{\prime}_{j} } } \right)f^{\prime}_{i} \hfill \\ + \left( {3\Lambda_{1} \sum\limits_{j = 0}^{i - 1} {f^{\prime}_{j} } \sum\limits_{n = 0}^{i - 1} {f^{\prime\prime}_{j} - \Lambda_{1} \sum\limits_{j = 0}^{i - 1} {f_{j} } \sum\limits_{j = 0}^{i - 1} {f^{\prime\prime\prime}_{j} + } } \sum\limits_{j = 0}^{i - 1} {f^{\prime\prime}_{j} } } \right)f_{i} = r_{1,i - 1} \hfill \\ \end{gathered}$$

25 $$\begin{gathered} \left( {\frac{1}{\Pr } - \frac{{\Lambda_{2} }}{2}\left( {\sum\limits_{j = 0}^{i - 1} {f_{j} } } \right)^{2} } \right)\theta^{\prime\prime}_{i} + \left( {\sum\limits_{j = 0}^{i - 1} {f_{j} } + \frac{{\Lambda_{2} }}{2}\left( {1 + 2A} \right)\sum\limits_{j = 0}^{i - 1} {f_{j} } \sum\limits_{j = 0}^{i - 1} {f^{\prime}_{j} } } \right)\theta^{\prime}_{i} \hfill \\ - \left( {\frac{{\Lambda_{2} }}{2}A\left( {2 + A} \right)\left( {\sum\limits_{j = 0}^{i - 1} {f^{\prime}_{j} } } \right)^{2} + A\sum\limits_{j = 0}^{i - 1} {f_{j} } - \frac{{\Lambda_{2} }}{2}A\sum\limits_{j = 0}^{i - 1} {f_{j} } \sum\limits_{j = 0}^{i - 1} {f^{\prime\prime}_{j} } } \right)\theta_{i} + \frac{{\Lambda_{2} }}{2}Af^{\prime\prime}_{i} \,\sum\limits_{j = 0}^{i - 1} {f_{j} } \sum\limits_{j = 0}^{i - 1} {\theta_{j} \,} \hfill \\ + \left( {\frac{{\Lambda_{2} }}{2}\left( {1 + 2A} \right)\sum\limits_{j = 0}^{i - 1} {f_{j} } \sum\limits_{j = 0}^{i - 1} {\theta^{\prime}_{j} } - A\sum\limits_{j = 0}^{i - 1} {\theta_{j} \,} - \Lambda_{2} \left( {2 + A} \right)A\sum\limits_{j = 0}^{i - 1} {f^{\prime}_{j} } \sum\limits_{j = 0}^{i - 1} {\theta_{j} \,} } \right)f^{\prime}_{i} \hfill \\ + \left( {\sum\limits_{j = 0}^{i - 1} {\theta^{\prime}_{j} } - \Lambda_{2} \sum\limits_{j = 0}^{i - 1} {f_{j} } \sum\limits_{j = 0}^{i - 1} {\theta^{\prime\prime}_{j} } + \frac{{\Lambda_{2} }}{2}\left( {1 + 2A} \right)\sum\limits_{j = 0}^{i - 1} {f_{j} } \sum\limits_{j = 0}^{i - 1} {\theta^{\prime}_{j} } + \frac{{\Lambda_{2} }}{2}A\sum\limits_{j = 0}^{i - 1} {f^{\prime\prime}_{j} } \sum\limits_{j = 0}^{i - 1} {\theta_{j} } } \right)f_{i} = r_{2,i - 1} \hfill \\ \end{gathered}$$

26 $$\phi^{\prime\prime}_{i} + S_{c} \phi^{\prime}_{i} \sum\limits_{j = 0}^{i - 1} {f_{j} } \, - S_{c} \phi_{i} \sum\limits_{j = 0}^{i - 1} {f^{\prime}_{j} } \, - S_{c} f^{\prime}_{i} \sum\limits_{j = 0}^{i - 1} {\phi_{j} } \, + S_{c} f_{i} \sum\limits_{j = 0}^{i - 1} {\phi^{\prime}_{j} } \, = r_{3,i - 1} \,,$$

depending on the conditions at the boundary,

$$\begin{gathered} f_{i} (0) = \,f^{\prime}_{i} (0) = f^{\prime}_{i} (\infty ) = 0,\,\,\theta_{i} (0) = \,\theta_{i} (\infty ) = 0,\, \hfill \\ \,\phi_{i} (0) = \,\phi_{i} (\infty ) = 0\,\,. \hfill \\ \end{gathered}$$

where $$r_{1,i - 1} = \left( {\frac{{\Lambda_{1} }}{2}\left( {\sum\limits_{j = 0}^{i - 1} {f_{j} } } \right)^{2} - 1} \right)\sum\limits_{j = 0}^{i - 1} {f^{\prime\prime\prime}_{j} } + 2\left( {\sum\limits_{j = 0}^{i - 1} {f^{\prime}_{j} } } \right)^{2} + 2\Lambda_{1} \left( {\sum\limits_{j = 0}^{i - 1} {f^{\prime}_{j} } } \right)^{3} \,$$

$$\,\,\,\, - \left( {\sum\limits_{j = 0}^{i - 1} {f_{j} } + \frac{\eta }{2}\Lambda_{1} \left( {\sum\limits_{j = 0}^{i - 1} {f^{\prime}_{j} } } \right)^{2} + 3\Lambda_{1} \sum\limits_{j = 0}^{i - 1} {f_{n} } \sum\limits_{j = 0}^{i - 1} {f^{\prime}_{j} } } \right)\sum\limits_{j = 0}^{i - 1} {f^{\prime\prime}_{j} }$$

$$\begin{gathered} r_{2,i - 1} = - \frac{1}{\Pr }\sum\limits_{j = 0}^{i - 1} {\theta^{\prime\prime}_{j} \,} + \frac{{\Lambda_{2} }}{2}\left( {\sum\limits_{j = 0}^{i - 1} {f_{j} } } \right)^{2} \sum\limits_{j = 0}^{i - 1} {\theta^{\prime\prime}_{j} \,} - \sum\limits_{j = 0}^{i - 1} {f_{j} } \sum\limits_{j = 0}^{i - 1} {\theta^{\prime}_{j} \,} \,\, - \frac{{\Lambda_{2} }}{2}\left( {1 + 2A} \right)\sum\limits_{j = 0}^{i - 1} {f_{j} } \sum\limits_{j = 0}^{i - 1} {f^{\prime}_{j} \sum\limits_{j = 0}^{i - 1} {\theta^{\prime}_{j} \,} } \hfill \\ \,\,\,\,\,\,\,\,\,\,\,\,\, - \frac{{\Lambda_{2} }}{2}A\left( {2 + A} \right)\left( {\sum\limits_{j = 0}^{i - 1} {f^{\prime}_{j} } } \right)^{2} \sum\limits_{j = 0}^{i - 1} {\theta_{j} \,} + A\sum\limits_{j = 0}^{i - 1} {f_{j} } \sum\limits_{j = 0}^{i - 1} {\theta_{j} \,} - \frac{{\Lambda_{2} }}{2}A\sum\limits_{j = 0}^{i - 1} {f_{j} } \sum\limits_{j = 0}^{i - 1} {f^{\prime\prime}_{j} } \sum\limits_{j = 0}^{i - 1} {\theta_{j} \,} \hfill \\ \,\,\,\,\,\,\,\,\,\,\,\, \hfill \\ \end{gathered}$$

$$r_{3,i - 1} = - \sum\limits_{j = 0}^{i - 1} {\phi^{\prime\prime}_{j} } - S_{c} \sum\limits_{j = 0}^{i - 1} {f_{j} } \,\sum\limits_{j = 0}^{i - 1} {\phi^{\prime}_{j} } + S_{c} \sum\limits_{j = 0}^{i - 1} {f^{\prime}_{j} } \,\sum\limits_{j = 0}^{i - 1} {\phi_{j} }$$

Using the Chebyshev collocation spectral method^43^, the linearized system was solved, resulting in the system of equations below:

27 $$\begin{gathered} A_{11} \,f_{i} + A_{12} \,\theta_{i} + A_{13} \,\phi_{i} = r_{1,i - 1} \hfill \\ A_{21} \,f_{i} + A_{22} \,\theta_{i} + A_{23} \,\phi_{i} = r_{2,i - 1} \hfill \\ A_{31} \,f_{i} + A_{32} \,\theta_{i} + A_{33} \,\phi_{i} = r_{3,i - 1} \hfill \\ \end{gathered}$$

We can write system (27) as matrix equation as

28 $$A_{i - 1} X_{i} = R_{i - 1} ,$$

where $$A_{i - 1} = \left[ {\begin{array}{*{20}c} {A_{11} } & {A_{12} } & {A_{13} } \\ {A_{21} } & {A_{22} } & {A_{23} } \\ {A_{31} } & {A_{32} } & {A_{33} } \\ \end{array} } \right],\,\,X_{i} = \left[ {\begin{array}{*{20}c} {f_{i} } \\ {\theta_{i} } \\ {\phi_{i} } \\ \end{array} } \right],\,\,R_{i - 1} = \left[ {\begin{array}{*{20}c} {r_{1,i - 1} } \\ {r_{2,i - 1} } \\ {r_{3,i - 1} } \\ \end{array} } \right]\,,$$

$$\begin{gathered} A_{11} = \left( {1 - \frac{{\Lambda_{1} }}{2}\left( {\sum\limits_{j = 0}^{i - 1} {f_{j} } } \right)^{2} } \right)D^{3} + \left( {\sum\limits_{j = 0}^{i - 1} {f_{j} } + \frac{\eta }{2}\Lambda_{1} \left( {\sum\limits_{j = 0}^{i - 1} {f^{\prime}_{j} } } \right)^{2} + 3\Lambda_{1} \sum\limits_{j = 0}^{i - 1} {f_{j} } \sum\limits_{j = 0}^{i - 1} {f^{\prime}_{j} } } \right)D^{2} \,\, \hfill \\ \,\,\,\,\,\,\,\,\,\,\,\, + \left( {3\Lambda_{1} \sum\limits_{j = 0}^{i - 1} {f_{j} } \sum\limits_{j = 0}^{i - 1} {f^{\prime\prime}_{j} + \eta \Lambda_{1} \sum\limits_{j = 0}^{i - 1} {f^{\prime}_{j} } \sum\limits_{j = 0}^{i - 1} {f^{\prime\prime}_{j} - 6} } \Lambda_{1} \left( {\sum\limits_{j = 0}^{i - 1} {f^{\prime}_{j} } } \right)^{2} - 4\sum\limits_{j = 0}^{i - 1} {f^{\prime}_{j} } } \right)D \hfill \\ \,\,\,\,\,\,\,\,\,\,\, + \left( {3\Lambda_{1} \sum\limits_{j = 0}^{i - 1} {f^{\prime}_{j} } \sum\limits_{j = 0}^{i - 1} {f^{\prime\prime}_{j} - \Lambda_{1} \sum\limits_{j = 0}^{i - 1} {f_{j} } \sum\limits_{j = 0}^{i - 1} {f^{\prime\prime\prime}_{j} + } } \sum\limits_{j = 0}^{i - 1} {f^{\prime\prime}_{j} } } \right), \hfill \\ \end{gathered}$$

$$A_{12} = A_{13} = A_{23} = A_{32} = 0\,\,,$$

$$\begin{gathered} A_{21} = \frac{{\Lambda_{2} }}{2}A\sum\limits_{j = 0}^{i - 1} {f_{j} } \sum\limits_{j = 0}^{i - 1} {\theta_{j} \,} D^{2} + \left( {\frac{{\Lambda_{2} }}{2}\left( {1 + 2A} \right)\sum\limits_{j = 0}^{i - 1} {f_{j} } \sum\limits_{j = 0}^{i - 1} {\theta^{\prime}_{j} } - A\sum\limits_{j = 0}^{i - 1} {\theta_{j} \,} } \right.\,\left. { - \Lambda_{2} \left( {2 + A} \right)A\sum\limits_{j = 0}^{i - 1} {f^{\prime}_{j} } \sum\limits_{j = 0}^{i - 1} {\theta_{j} \,} } \right)D \hfill \\ + \left( {\sum\limits_{j = 0}^{i - 1} {\theta^{\prime}_{j} } - \Lambda_{2} \sum\limits_{j = 0}^{i - 1} {f_{j} } \sum\limits_{j = 0}^{i - 1} {\theta^{\prime\prime}_{j} } } \right.\,\left. { + \frac{{\Lambda_{2} }}{2}\left( {1 + 2A} \right)\sum\limits_{j = 0}^{i - 1} {f_{j} } \sum\limits_{j = 0}^{i - 1} {\theta^{\prime}_{j} } + \frac{{\Lambda_{2} }}{2}A\sum\limits_{j = 0}^{i - 1} {f^{\prime\prime}_{j} } \sum\limits_{j = 0}^{i - 1} {\theta_{j} } } \right), \hfill \\ \end{gathered}$$

$$A_{31} = - S_{c} \sum\limits_{j = 0}^{i - 1} {\phi_{j} } \,D + S_{c} \sum\limits_{j = 0}^{i - 1} {\phi^{\prime}_{j} } \,\,,\,\,\,\,A_{33} = D^{2} + S_{c} \sum\limits_{j = 0}^{i - 1} {f_{j} } \,D - S_{c} \sum\limits_{j = 0}^{i - 1} {f^{\prime}_{j} } \,\,.\,\,\,$$

The resultant system (28) is readily solved as

29 $$X_{i} = A_{i - 1}^{ - 1} \,R_{i - 1}$$

## Results and discussion

This paper analyzed the effects of chemical reaction on the Cattaneo–Christov heat flux model for viscoelastic flow over an exponentially stretching sheet. Transfigured governing Eqs. (18) – (20) with the boundary conditions (21) are coupled non-linear differential equations. Thus, it is impossible to solve directly with the analytical method. Therefore, to solve this coupled non-linear differential equations, we use SLM (SLM) method by MatLabR2023a software. For various values of effective governing parameters such as velocity ratio U, Deborah number $$\Lambda_{1}$$, Prandtl number $$\Pr$$, Schmidt number $$S_{c}$$, reaction rate parameter $$\gamma$$, and thermal relaxation time $$\Lambda_{2}$$, the numerical solutions of velocity, temperature, and concentration are obtained. The convergence of SLM solutions with respect to several orders of approximations for $$- f^{\prime\prime}(0)$$,$$- \theta^{\prime}(0)$$ and $$- \phi^{\prime}(0)$$ for different values of $$\Lambda_{1}$$ when $$\Lambda_{2} = 0.5,\,A = 1.5,\,$$$$\gamma = 1,\,S_{c} = 0.2,\,\Pr = 1,$$ is presented in Table 1. The comparison of the variation of the Nusselt number $$- \theta^{\prime}(0)$$ for different values of $$\Lambda_{1}$$ is presented in Table 2. The values show that our result is in admirable agreement with the results given by researchers Khan^6^ in limiting conditions. Moreover, a comparison of different values of the Prandtl number $$\Pr$$ in the event that $$\Lambda_{1} = \Lambda_{2} = 0$$, as well as the local Nusselt number $$\theta^{\prime}_{{}} (0)$$ for a range of parameter values are shown in Table 3. It can be observed that when the Prandtl number $$\Pr$$ and the parameter $$A$$ are increased, the local Nusselt number $$\theta^{\prime}_{{}} (0)$$ also grows in magnitude. Furthermore, it has been discovered that there is a strong agreement between the current numerical values of the local Nusselt number $$\theta^{\prime}_{{}} (0)$$ and the numerical outcomes covered by Magyari and Keller^32^. Therefore, we are assured that for the analysis of our problem, the numerical method is appropriate. The SLM findings for the local Nusselt number, local Sherwood number, and skin friction coefficient are shown in Table 4 for various parameter values.

**Table 1:** Values of $$- f^{\prime\prime}(0)$$,$$- \theta^{\prime}(0)$$ and $$- \phi^{\prime}(0)$$ for different values of $$\Lambda_{1}$$ when $$\Lambda_{2} = 0.5,\,A = 1.5,\,$$$$\gamma = 1,\,S_{c} = 0.2,\,\Pr = 1.$$

$\Lambda_{1}$	1st iteration	2nd iteration	3rd iteration	4th iteration	5th iteration
$$- f^{\prime\prime}(0)$$					
0.0	1.281802692	1.281808558	1.281808558	1.281808558	1.281808558
0.5	1.560862928	1.560888965	1.560888970	1.560888970	1.560888970
1.0	1.796477879	1.796488299	1.796488300	1.796488300	1.796488300
1.5	2.004291165	2.004296302	2.004296302	2.004296302	2.004296302
$$- \theta^{\prime}(0)$$					
0.0	1.550676673	1.550973007	1.550973582	1.550973582	1.550973582
0.5	1.463275123	1.463631137	1.463635942	1.463635942	1.463635942
1.0	1.395076645	1.395373349	1.395375811	1.395375812	1.395375812
1.5	1.338887820	1.339125712	1.339127152	1.339127152	1.339127152
$$- \phi^{\prime}(0)$$					
0.0	0.589426648	0.589423996	0.589423996	0.589423996	0.589423996
0.5	0.577328012	0.577319150	0.577319148	0.577319148	0.577319148
1.0	0.568742024	0.568738456	0.568738455	0.568738455	0.568738455
1.5	0.562136664	0.562134897	0.562134897	0.562134897	0.562134897

**Table 2:** Values of $\theta^{\prime}(0)$ for different values of $\Pr ,\,\Lambda_{1} ,\,\Lambda_{2} \,$ and $A$ compared to previous results Khan et al.^6^.

$\Pr$	$\Lambda_{1}$	$\Lambda_{2}$	$- \theta^{\prime}(0)$					
			$A = - 1.5$ Khan et al.^6^	$A = - 1.5$ Present	$A = 0$ Khan et al.^6^	$A = 0$ Present	$A = 1.5$ Khan et al.^6^	$A = 1.5$ Present
1	0.5	0	0.333441	0.335535	− 0.512599	− 0.512080	− 1.06969	− 1.06953
		0.5	0.532307	0.533327	− 0.570367	− 0.570211	− 1.46365	− 1.46364
		1	0.755562	0.755901	− 0.635466	− 0.635459	− 1.82605	− 1.82606
	0	0.5	0.608711	0.608883	− 0.622927	− 0.622929	− 1.55096	− 1.55097
	0.5		0.532307	0.533324	− 0.570367	− 0.570211	− 1.46365	− 1.46364
	1		0.480170	0.482886	− 0.532685	− 0.532085	− 1.39552	− 1.39538

**Table 3:** Values of the $\theta^{\prime}(0)$ for different values of $\Pr$ and $A$ when $\,\Lambda_{1} = 0\,\,{\text{and}}\,\Lambda_{2} = 0\,$. The values in the brackets are from Magyari and Keller^36^.

$$A$$ \$$\Pr$$	0.5	1	3	5	8	10
− 1.5	0.204048 (0.204049)	0.377413 (0.377413)	0.923857 (0.923857)	1.353241 (1.353240)	1.888492 (1.888500)	2.200028 (2.200000)
− 0.5	− 0.175816 (− 0.175815)	− 0.299877 (− 0.299876)	− 0.634111 (− 0.634113)	− 0.870428 (− 0.870431)	− 1.150317 (− 1.150321)	− 1.308609 (− 1.308613)
0	− 0.330494 (− 0.330493)	− 0.549644 (− 0.549643)	− 1.122087 (− 1.122188)	− 1.521239 (− 1.521243)	− 1.991838 (− 1.991847)	− 2.257424 (− 2.257429)
1	− 0.594339 (− 0.594338)	− 0.954783 (− 0.954782)	− 1.869074 (− 1.869075)	− 2.500132 (− 2.500135)	− 3.242119 (− 3.242129)	− 3.660372 (− 3.660379)
3	− 1.008408 (− 1.008405)	− 1.560295 (− 1.560294)	− 2.938534 (− 2.938535)	− 3.886556 (− 3.886555)	− 5.000464 (− 5.000465)	− 5.628196 (− 5.628198)

**Table 4:** Using SLM, the effects of skin friction, the local Nusselt number, and the local Sherwood number were calculated for various parameter values.

$\Pr$	$S_{c}$	$\gamma$	$\Lambda_{1}$	$\Lambda_{2}$	$A$	$- f^{\prime\prime}(0)$	$- \theta^{\prime}(0)$	$- \phi^{\prime}(0)$
0.7	0.2	1	0.5	0.5	1.5	1.560888970	1.143009943	0.577319148
1						1.560888970	1.463635942	0.577319148
1.5						1.560888970	1.911146538	0.577319148
2						1.560888970	2.291308778	0.577319148
1	0.2	1	0.5	0.5	1.5	1.560888970	1.463635942	0.577319148
	0.3					1.560888970	1.463635942	0.724565301
	0.4					1.560888970	1.463635942	0.850847360
	0.5					1.560888970	1.463635942	0.963353025
1	0.2	1	0.5	0.5	1.5	1.560888970	1.463635942	0.577319148
		2				1.560888970	1.463635942	0.743605534
		3				1.560888970	1.463635942	0.874728532
		5				1.560888970	1.463635942	1.086651111
1	0.2	1	0.0	0.5	1.5	1.281808558	1.550973582	0.589423996
			0.5			1.560888970	1.463635942	0.577319148
			1.0			1.796488300	1.395375812	0.568738455
			1.5			2.004296302	1.339127152	0.562134897
1	0.2	1	0.5	0.0	1.5	1.560888970	1.069530700	0.577319148
				0.5		1.560888970	1.463635942	0.577319148
				1.0		1.560888970	1.826064982	0.577319148
				1.5		1.560888970	2.160677592	0.577319148
1	0.2	1	0.1	0.5	-1.5	1.560888970	-0.533323613	0.577319148
					0.0	1.560888970	0.570210705	0.577319148
					1.5	1.560888970	1.463635942	0.577319148
					3.0	1.560888970	2.285200915	0.577319148

**Figure 2:**
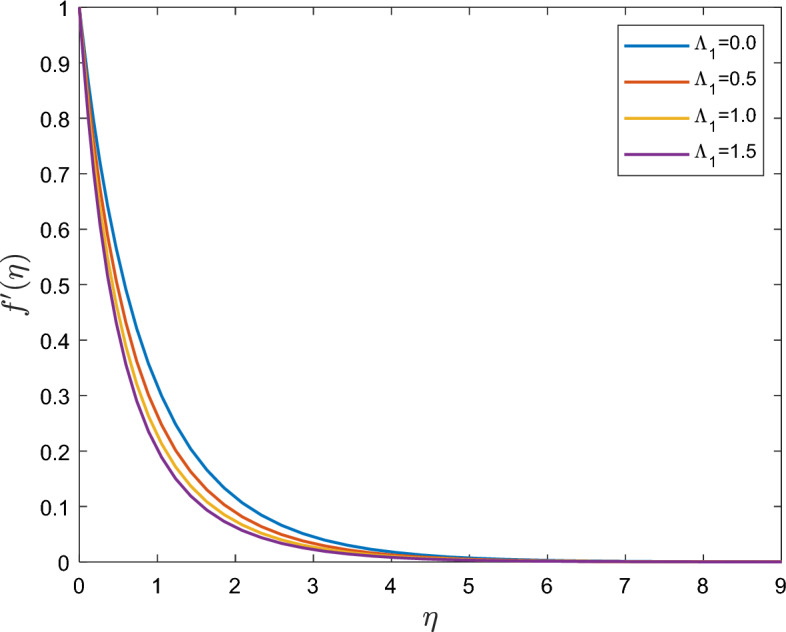
Effect of the relaxation time $\Lambda_{1}$ on $f^{\prime}(\eta )$.

Figure 2 shows what happens to the hydrodynamic boundary layer when a fluid has a nondimensional relaxation time. An increase in $\Lambda_{1}$ is interpreted as an increase in fluid viscosity. The fluid motion is resisted by increasing viscosity, which causes the velocity to decrease. Given that the Deborah number $\Lambda_{1}$ is a good indicator of how long it will take a fluid to relax and come to rest when shear tension is eliminated, the thickness of the boundary layer likewise decreases for large $\Lambda_{1}$ values. Many polymeric liquids that defy the Newtonian fluid model display these kinds of behaviors. The flow between two neighboring layers will decrease with an increase in Deborah number. Velocity and boundary layer thickness are generally reduced as a result.

**Figure 3:**
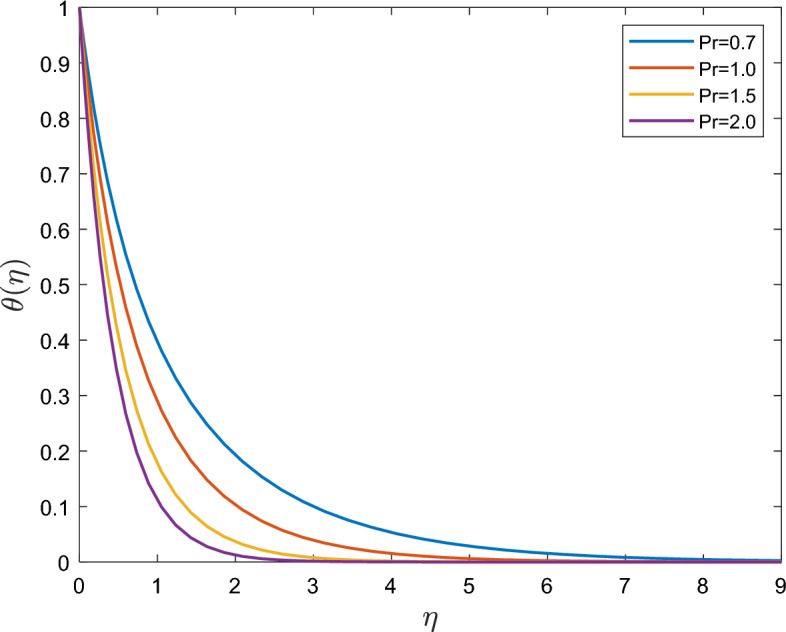
Effect of the Prandtl number $\Pr$ of on $\theta (\eta )$.

Figure 3 illustrates the changes in Prandtl number $\Pr$ when considering the thermal relaxation time. With rising $\Pr$, the thermal boundary layer's thickness and temperature decrease. which is qualitatively identical to the behavior of $\Pr$ on $\theta$ in both scenarios. In particular, the temperature variations of both the Fourier and Cattaneo–Christov heat flux models have the same value as that of $\theta$. Physically, the thermal diffusivity $\alpha$ and Prandtl number $\Pr$ are inversely correlated. The fluid is thought to experience less thermal influence as $\Pr$ increases. Therefore, when $\Pr$ increases, the thermal boundary layer becomes thinner. Owing to the thinner thermal boundary layer, the temperature profile is steeper, indicating that the wall slope of the temperature function is greater.

**Figure 4:**
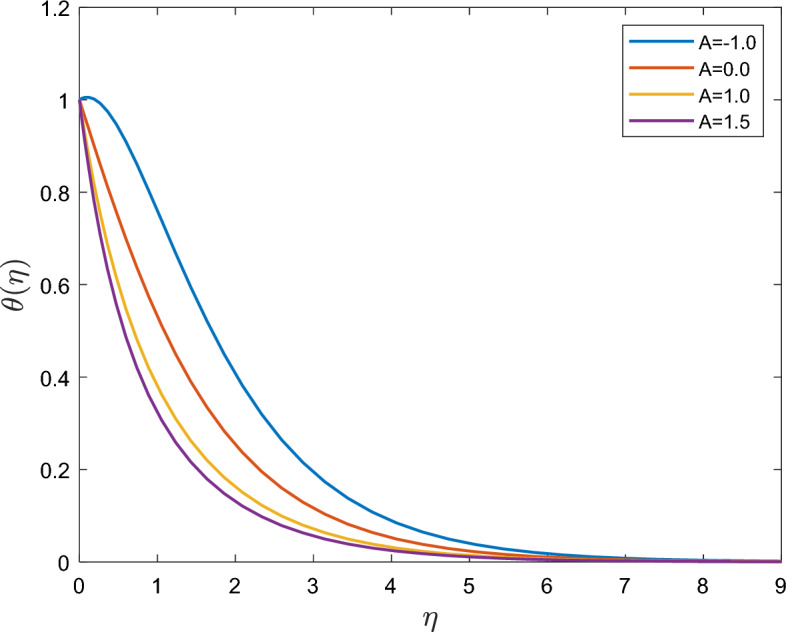
Effect of temperature exponent $A$ on $\theta (\eta )$.

The effect of the temperature exponent $A$ on the temperature profile is illustrated in Fig. 4. This figure shows the interesting ‘Sparrow-Gregg hill’ (SGH) phenomenon, in which temperature increases first reach their highest point before falling exponentially to zero. This implies that, for some negative reasons, reverse heat flow towards the sheet should be expected. The wall slope of the temperature function increased sharply as the positive/negative temperature exponent parameter $A$ increased.

**Figure 5:**
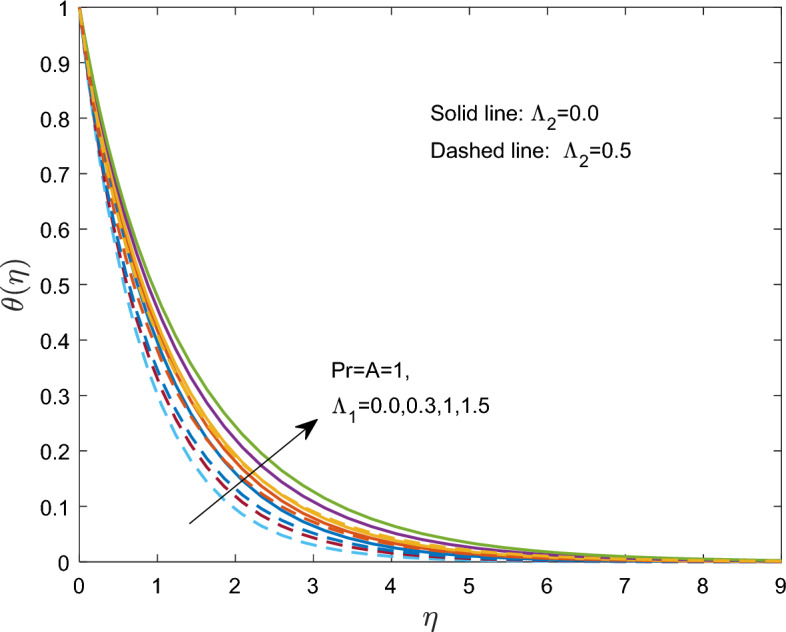
Effect of the relaxation time $\Lambda_{1}$ of on $\theta (\eta )$.

The effect of $\Lambda_{1}$ on the thermal boundary layer is shown in Fig. 5. A larger $\Lambda_{1}$ produces a stronger viscous force that resists the flow and raises the temperature. As a result, viscoelastic fluid has a higher temperature than a viscous fluid.

**Figure 6:**
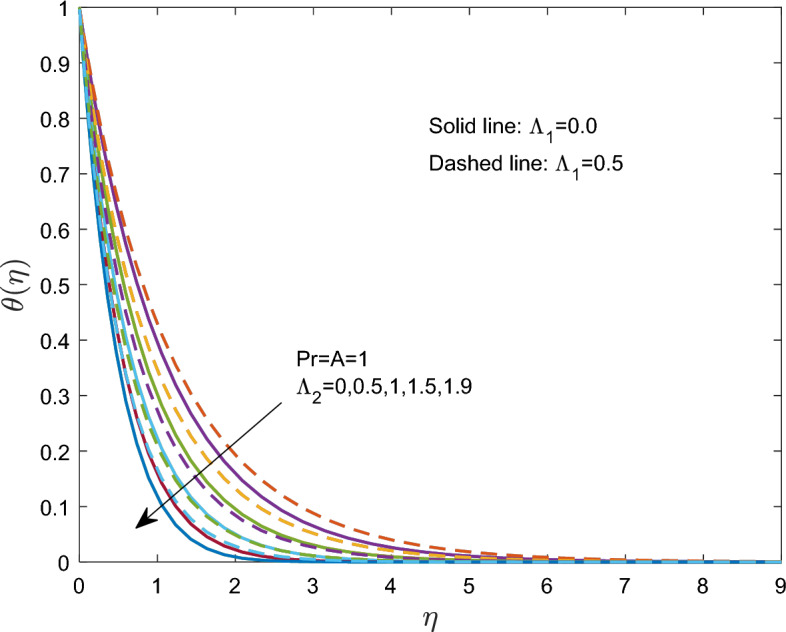
Effect of thermal relaxation time $\Lambda_{2}$ on $\theta (\eta )$.

Figure 6 shows how the temperature distribution is affected by the nondimensional thermal relaxation time $\Lambda_{2}$. The thermal relaxation time and $\theta$ temperature have an inverse relationship. The temperature $\theta$ approached the free-stream condition at closer ranges above the sheet as $\Lambda_{2}$ increased. In particular, both Newtonian and Maxwell fluids exhibit similar magnitudes of temperature $\theta$ changes with the thermal relaxation time.

**Figure 7:**
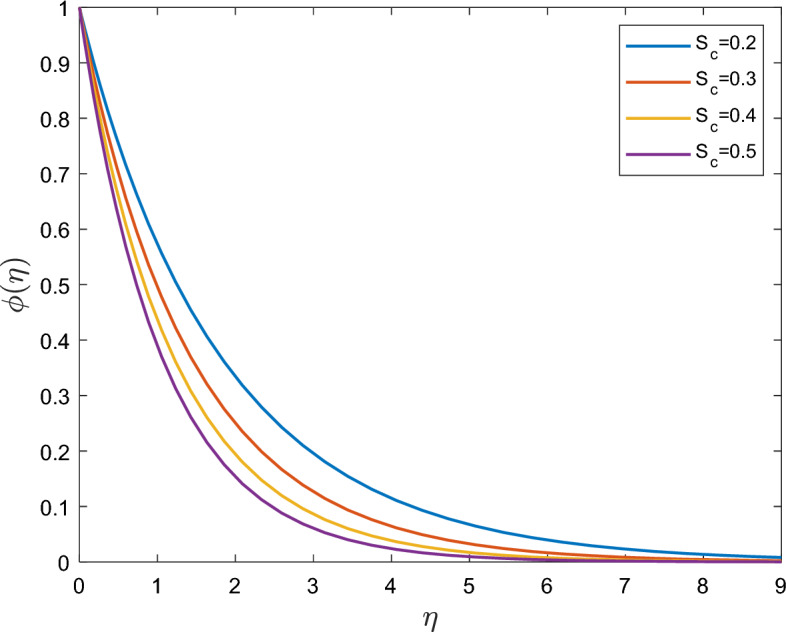
Effect of the Schmidt number $S_{c}$ on $\phi (\eta )$.

Figure 7 shows that a decrease in concentration has been associated with an increase in Schmidt number $S_{c}$. A lower mass diffusivity is associated with a lower Schmidt number $S_{c}$. This elucidates why the thickness of the boundary layer concentration decreases as $S_{c}$ increases.

**Figure 8:**
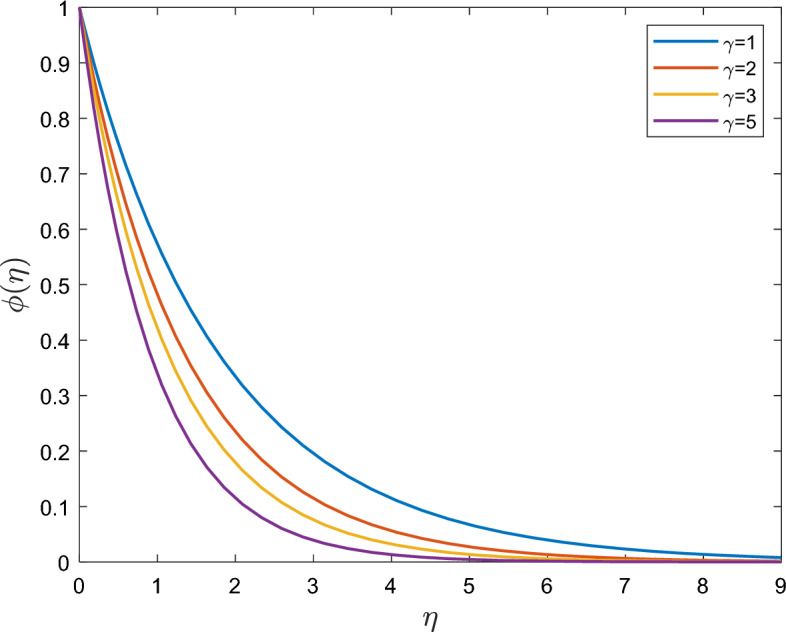
Effect of reaction rate $\gamma$ on $\phi (\eta )$.

Figure 8 shows how a change in the reaction rate parameter $\gamma$ affects the concentration profile. We observe that there is a noticeable decrease in concentration with an increase in $\gamma$. The contour in the free flow is uniformly attenuated to a static value after the velocity climbs noticeably close to the wall. As a result, the chemical reaction speeds up the flow or increases the instantaneous transfer. The concentration boundary layer becomes thinner as the level of $\gamma$ increase.

## Conclusions

In this study, the impact of chemical reaction on the Cattaneo–Christov heat flux model for viscoelastic flow over an exponentially stretching sheet was investigated. governing system of nonlinear PDEs is transformed into a system of nonlinear ODEs using appropriate similarity transformations. The converted system equations were solved using SLM. The numerical results obtained agree very well with previously reported cases available in the literature. The following is a summary of the study's main findings:

- In viscoelastic fluids, the hydrodynamic boundary layer is thinner than in viscous fluids.
- The thermal boundary layer thickness and temperature are decreasing functions of the relaxation time $\Lambda_{2}$.
- For negative temperature exponent A, there are interesting Sparrow-Gregg Hills (SGH) for the temperature distribution.
- Fourier's heat conduction law and the Cattaneo–Christov model's parameter responses are qualitatively comparable.
- The concentration boundary layer becomes thinner as the levels of Schmidt number $S_{c}$ and reaction rate $\gamma$ increase.
- By setting $\Lambda_{1} = 0$, we can retrieve the current consideration for the Newtonian fluid case.
- A few SLM iterations were enough to achieve great agreement with previous results.

## Data Availability

All data generated or analyzed during the study are included in this article.

## List of symbols

$$\left( {x,y} \right)$$: Cartesian coordinates [m]
$\left( {u,v} \right)$: Velocity components [ms^-1^]
$$\upsilon$$: Kinematic viscosity [m^2^s^-1^]
$\rho$: Density of fluid [Kg m^-1^]
$q$: The heat flux
$\gamma$: Chemical reaction rate
$$k$$: Thermal conductivity
$\lambda_{1}$: Fluid relaxation time
$\lambda_{2}$: Heat flow relaxation time.
$$T$$: Fluid Temperature [K]
$$\alpha$$: Thermal diffusivity [m^2^s^-1^]
$\rho c$: Fluid capacity heat [J m^-3^ K^-1^]
$c_{p}$: Specific heat [J/kg K]
$$D$$: Diffusion coefficient
$$L$$: Reference length [m]
$$A$$: Temperature exponent
$$P_{r}$$: Prandtl number
$$S_{c}$$: Schmidt number
$\Lambda_{1}$: Non-dimensional fluid relaxation times
$\Lambda_{2}$: Non-dimensional thermal relaxation times
$C_{w}$: Concentration field at the surface [mol]
$C$: Concentration of field [mol]
$C_{\infty }$: Ambient concentration field [mol]
$U_{w}$: Velocity at the wall [ms^-1^]
$$T_{w}$$: Surface temperature [K]
$$T_{\infty }$$: Ambient temperature [K

## References

[1] Fourier, J. B. J. Théorie analytique de la chaleur, Paris, (1822).

[2] Cattaneo C (1948). Sulla conduzione del calore. Atti Sem. Mat. Fis. Univ. Modena..

[3] Straughan B (2010). Thermal convection with the Cattaneo–Christov model. Int. J. Heat Mass Transfer..

[4] Ciarletta M, Straughan B (2010). Uniqueness and structural stability for the Cattaneo–Christov equations. Mech. Res. Commun..

[5] Mustafa M (2015). Cattaneo-Christov heat flux model for rotating flow and heat transfer of upper-convected Maxwell fluid. Aip Adv..

[6] Khan JA, Mustafa M, Hayat T, Alsaedi A (2015). Numerical study of Cattaneo–Christov heat flux model for viscoelastic flow due to an exponentially stretching surface. PLOS one.

[7] Sohail M, Naz R (2020). Modified heat and mass transmission models in the magnetohydrodynamic flow of Sutterby nanofluid in stretching cylinder. Phys. A Stat. Mech. Appl..

[8] Khan MI, Alzahrani F (2020). Cattaneo–Christov double diffusion (CCDD) and magnetized stagnation point flow of non-Newtonian fluid with internal resistance of particles. Phys. Scripta..

[9] Dadheech A, Parmar A, Agrawal K, Al-Mdallal Q, Sharma S (2022). Second law analysis for MHD slip flow for Williamson fluid over a vertical plate with Cattaneo–Christov heat flux. Case Stud. Therm. Eng..

[10] Mehmood Y, Sagheer M, Hussain S, Bilal M (2018). MHD stagnation point flow and heat transfer in viscoelastic fluid with Cattaneo–Christov heat flux model. Neural Comput. Appl..

[11] Hayat T, Khan SA, Khan MI, Momani S, Alsaedi A (2020). Cattaneo–Christov (CC) heat flux model for nanomaterial stagnation point flow of Oldroyd-B fluid. Comput. Methods Progr. Biomed..

[12] Ahmad S, Nadeem S, Muhammad N, Khan MN (2021). Cattaneo–Christov heat flux model for stagnation point flow of micropolar nanofluid toward a nonlinear stretching surface with slip effects. J. Therm. Anal. Calorimet..

[13] Naveen Kumar R, Suresh Goud J, Srilatha P, Manjunatha PT, Rani SP, Kumar R, Suresha (2022). Cattaneo-Christov heat flux model for nanofluid flow over a curved stretching sheet: An application of Stefan blowing. Heat Transfer..

[14] Shah Z, Rooman M, Shutaywi M (2023). Computational analysis of radiative engine oil-based Prandtl-Eyring hybrid nanofluid flow with variable heat transfer using the Cattaneo–Christov heat flux model. RSC Adv..

[15] Zeb S, Ullah Z, Urooj H, Khan I, Ganie AH, Eldin SM (2023). Simultaneous features of MHD and radiation effects on the UCM viscoelastic fluid through a porous medium with slip conditions. Case Stud. Therm. Eng..

[16] Salah F, Sidahmed AOM (2022). Chemical reaction and radiation effects on MHD flow of Oldroyd-B Fluid through porous medium past an Exponentially Stretching Sheet with Heat Sink. J. Appl. Math..

[17] Sidahmed A, Salah F (2022). Radiation effects on MHD flow of second grade fluid through porous medium past an exponentially stretching sheet with chemical reaction. J. Adv. Res. Fluid Mech. Therm. Sci..

[18] Seini YI, Makinde OD (2013). MHD boundary layer flow due to exponential stretching surface with radiation and chemical reaction. Math. Probl. Eng..

[19] Paul A, Tusar KD (2023). Thermal and mass transfer aspects of MHD flow across an exponentially stretched sheet with chemical reaction. Int. J. Ambient Energy..

[20] Shafiq A, Çolak AB, Sindhu TN (2023). Development of an intelligent computing system using neural networks for modeling bioconvection flow of second-grade nanofluid with gyrotactic microorganisms. Numer. Heat Transfer Part B Fundamentals.

[21] Shafiq A, Çolak AB, Sindhu TN (2024). Comparative analysis to study the Darcy-Forchheimer Tangent hyperbolic flow towards cylindrical surface using artificial neural network: An application to Parabolic Trough Solar Collector. Math. Comput. Simul..

[22] Agarwal K, Baghel RS, Parmar A, Dadheech A (2024). Jeffery slip fluid flow with the magnetic dipole effect over a melting or permeable linearly stretching sheet. Int. J. Appl. Comput. Math..

[23] Chu YM, Khan MI, Abbas T, Sidi MO, Alharbi KAM, Alqsair UF, Malik MY (2022). Radiative thermal analysis for four types of hybrid nanoparticles subject to non-uniform heat source: Keller box numerical approach. Case Stud. Therm. Eng..

[24] Nazir U, Sohail M, Mukdasai K, Singh A, Alahmadi RA, Galal AM, Eldin SM (2022). Applications of variable thermal properties in Carreau material with ion slip and Hall forces towards cone using a non-Fourier approach via FE-method and mesh-free study. Front. Mater..

[25] Liu J, Nazir U, Sohail M, Mukdasai K, Singh A, Alanazi M, Chambashi G (2023). Numerical investigation of thermal enhancement using MoS2–Ag/C2H6O2 in Prandtl fluid with Soret and Dufour effects across a vertical sheet. AIP Adv..

[26] Suneetha, S., Wahidunnisa, L., Reddy, S. R. R., & Reddy, P. B. A. Entropy generation on the variable electric field and EMHD SWCNT-blood nanofluid with melting/non-melting heat transfer. *Proceedings of the Institution of Mechanical Engineers, Part E: Journal of Process Mechanical Engineering*. ***237***(6), 2314-2322 10.1177/09544089221140223 (2023).

[27] Sharma S, Dadheech A, Parmar A, Arora J, Al-Mdallal Q, Saranya S (2023). MHD micro polar fluid flow over a stretching surface with melting and slip effect. Sci. Rep..

[28] Agarwal V, Singh B, Nisar KS (2022). Numerical analysis of heat transfer in magnetohydrodynamic micropolar jeffery fluid flow through porous medium over a stretching sheet with thermal radiation. J. Therm. Anal. Calorimet..

[29] Goyal M, Sharma S (2023). Investigation of Oldroyd-B fluid flow and heat transfer over a stretching sheet with nonlinear radiation and heat source. Heat Transfer.

[30] Reddy SRR, Jakeer S, Rupa ML (2023). ANN model of three-dimensional micropolar dusty hybrid nanofluid flow with coriolis force: biomedical applications. Indian J. Phys..

[31] Makukula Z, Sibanda P, Motsa S (2010). A note on the solution of the von Kármán equations using series and Chebyshev spectral methods. Boundary Value Probl..

[32] Ahmed MAM, Mohammed EM, Khidir AA (2015). On linearization method to MHD boundary layer convective heat transfer with low pressure gradient. Propul. Power Res..

[33] Khidir AA (2023). Application of successive linearisation method on mixed convection boundary layer flow of nanofluid from an exponentially stretching surface with magnetic field effect. J. Nanofluids..

[34] Daoud Y, Mohammed A, Khidir AA (2021). On the solution of magneto-hydrodynamics three-dimensional flow due to a stretching sheet in a porous medium using the successive linearization method. Chin. J. Phys..

[35] Salah F, Alzahrani AK, Sidahmed AO, Viswanathan KK (2019). A note on thin-film flow of Eyring-Powell fluid on the vertically moving belt using successive linearization method. Int. J. Adv. Appl. Sci..

[36] Magyari E, Keller B (1999). Heat and mass transfer in the boundary layers on an exponentially stretching continuous surface. J. Phys. D Appl. Phys..

[37] Reddy NN, Rao VS, Reddy BR (2021). Chemical reaction impact on MHD natural convection flow through porous medium past an exponentially stretching sheet in presence of heat source/sink and viscous dissipation. Case Stud. Therm. Eng..

[38] Schlichting H, Kestin J (1961). Boundary layer theory.

[39] Dong Y, Cao BY, Guo ZY (2011). Generalized heat conduction laws based on thermomass theory and phonon hydrodynamics. J. Appl. Phys..

[40] Han S, Zheng L, Li C, Zhang X (2014). Coupled flow and heat transfer in viscoelastic fluid with Cattaneo–Christov heat flux model. Appl. Math. Lett..

[41] Christov CI (2009). On frame indifferent formulation of the Maxwell-Cattaneo model of finite-speed heat conduction. Mech. Res. Commun..

[42] Ahmed MAM, Mohammed ME, Khidir AA (2016). The effects of cross-diffusion and radiation on mixed convection from a vertical flat plate embedded in a fluid-saturated porous medium in the presence of viscous dissipation. Propul. Power Res..

[43] Hussaini MY, Zang TA (1987). Spectral methods in fluid dynamics. Ann. Rev. Fluid Mech..

